# Two Attacks of Scarlet Fever Occurring in the Same Patient within a Few Months

**Published:** 1901-03-09

**Authors:** Alfred Greenwood

**Affiliations:** Medical Officer of Health and Medical Superintendent to the Crewe Fever Hospital


### TWO ATTACKS OF SCARLET FEVER OCCURRING IN THE SAME PATIENT WITHIN A FEW MONTHS.

By ALFRED GREENWOOD, M.D., D.P.H., Medical Officer of Health and Medical Superintendent to the Crewe Fever Hospital.

THE following case is of sufficient interest to record:—

F. H. W., girl, aged 14 years, was admitted to the Crewe Fever Hospital on June 7th, 1900, with a scarlatinal rash and throat and a temperature of 100°·6 Fahr. The attack, although undoubtedly scarlatinal, was a mild one. She had never had any previous illness and was in good condition. The girl peeled well, had no albuminuria, aural or nasal discharge, and left the hospital quite well in six weeks.

The same girl was again admitted to hospital on November 27th, 1900, having a well-marked scarlatinal rash and throat and a temperature of 100°·8 Fahr. The symptoms were more marked this time, the attack being more severe than the first one. Desquamation proceeded more slowly than before, but the patient had no complications, and was discharged cured after she had been in the hospital rather more than seven weeks.

CONCLUSIONS.

1. This case, therefore, shows that one attack of scarlet fever does not confer immunity from the occurrence of a second attack within a few months.

2. The second attack was more severe than the first.

3. Although, generally speaking, the lessened liability with increasing age is greater than can be accounted for either by protection given by previous attacks or by diminished exposure to infection, and must be attributed to a real and increasing insusceptibility, there are exceptions to this.

4. The source of infection in each attack was probably the same, viz. an infected school, which I was compelled to close owing to the presence of several children in a state of scarlatinal desquamation.

5. The need for increased knowledge in the bacteriology of scarlet fever which would confirm the diagnosis of those cases which are of clinical interest, and which would greatly aid in the discrimination between this and other diseases.

